# Bioaerosols and Airway Diseases: Mechanisms of Epithelial Dysfunction, Immune Activation, and Strategies for Exposure Mitigation

**DOI:** 10.26502/aimr.0210

**Published:** 2025-07-03

**Authors:** Leena Nabipur, Michael Mouawad, Devendra K. Agrawal

**Affiliations:** 1 Department of Translational Research, College of Osteopathic Medicine of the Pacific, Western University of Health Sciences, Pomona, California 91766 USA.

**Keywords:** Airway disease, Airway epithelium, Allergic inflammation, Allergy, Asthma, Bio-aerosols, Genetic polymorphism, Immune activation, Innate immune response, Mucociliary clearance, Toll-like receptor

## Abstract

Bioaerosols—airborne particles of biological origin such as bacteria, fungi, viruses, and allergens—are increasingly recognized as critical environmental factors in the pathogenesis of airway diseases, particularly asthma. This article provides current understanding of how bioaerosols interact with the airway epithelium to initiate acute immune responses, promote chronic inflammation, and drive airway remodeling. Key mechanisms include disruption of mucociliary clearance, activation of innate immune receptors such as TLRs and PRRs, and the role of surfactant proteins SP-A and SP-D in modulating allergic inflammation. Chronic exposure leads to cytokine-mediated fibrosis and smooth muscle hypertrophy, contributing to steroid-resistant asthma. Genetic polymorphisms, especially in innate immunity genes like TLR2, TLR4, and CD14, influence individual susceptibility. The complexity of bioaerosol composition, coupled with environmental variability and lack of standardized exposure thresholds, presents challenges for effective monitoring. However, emerging strategies such as source control, improved ventilation, HEPA filtration, UV disinfection, and real-time airborne pathogen detection offer promising avenues for exposure mitigation. This comprehensive review underscores the need for interdisciplinary approaches to better understand and manage bioaerosol-related respiratory health risks.

## I. Introduction

Bioaerosols are airborne particles composed of biological substances such as bacteria, fungi, viruses, pollen, and other organic matter. These particles are ubiquitous in both indoor and outdoor environments, varying in size, composition, and viability. They can stem from a wide range of sources, both natural and anthropogenic.

Anthropogenic sources include industrial operations, agricultural activities, wastewater treatment facilities, and indoor environments like hospitals and homes. Occupational settings such as waste sorting facilities, composting plants, livestock farms, and food processing units are particularly high-risk, where frequent exposure by workers to elevated levels of bioaerosols have led to an increased incidence of respiratory conditions, including asthma, rhinitis, and chronic inflammation [1–3]. Indoors, bioaerosols contribute to 5–34% of particulate matter, entering through outdoor infiltration or arising from indoor sources such as building materials, furnishings, pets, plants, organic waste, and human activity—including coughing, sneezing, walking, and even toilet flushing [4–6].

Environmental factors such as temperature, humidity, and ventilation dynamics significantly influence microbial growth, aerosolization, and dispersion [7–11]. Climate change and urbanization further compound these effects, leading to altered bioaerosol distributions and increased exposure risks in densely populated regions [12–14]. As such, bioaerosols are increasingly recognized as key contributors to respiratory and systemic illnesses, including pneumonia, influenza, measles, asthma, allergies, and gastrointestinal infections [15].

Inhalation of bioaerosols can initiate immune responses that range from allergic sensitization to infection and chronic inflammation. Vulnerable populations—such as children, the elderly, and those with underlying respiratory or immune conditions—are disproportionately affected [16,17]. While the negative impacts are well documented, certain types of microbial exposures may be protective. For example, early-life exposure to diverse microbial environments has been associated with immune modulation and reduced risk of allergies and asthma, particularly in children raised in rural or farm settings [18].

Despite growing awareness of bioaerosol-associated health effects, there remains considerable uncertainty in defining causality and risk thresholds. Variability in microbial composition, inconsistencies in sampling and analysis methods, and a lack of standardized exposure metrics complicate efforts to establish clear dose-response relationships [19–25]. Moreover, individual susceptibility and the influence of co-exposures to non-biological environmental stressors further obscure the pathophysiological landscape.

Inhalation of bioaerosols trigger allergic reactions, airway inflammation, airway constriction, exacerbation of asthma symptoms, and other pulomonary infections [26–33]. Bioaerosols promote epithelial barrier dysfunction, oxidative stress, and dysregulated immune responses. Iqbal et al. conducted a systematic review and meta-analysis showing strong associations between bioaerosol exposure and respiratory disease, particularly in high-burden environments such as poultry farms, waste sites, composting facilities, and industrial plants [34]. These settings often contain high concentrations of pro-inflammatory components like endotoxins and fungal β-glucans, which activate Toll-like receptors (TLRs) and other pattern recognition receptors (PRRs) on epithelial and immune cells. This interaction leads to cytokine release (e.g., IL-6, TNF-α, IL-8), immune cell recruitment, and chronic inflammation [35–37]. Repeated or prolonged exposure in such environments can impair lung function, drive bronchial hyperresponsiveness, and lead to airway remodeling.

In addition, emerging research suggests that the composition of the airway microbiome may influence host susceptibility to bioaerosol-induced inflammation. Dysbiosis—disruption of the normal microbial flora—may exacerbate responses to inhaled pathogens or allergens, compounding the risk of chronic respiratory disease.

Understanding these mechanisms is critical for developing effective prevention and mitigation strategies. Current research focuses on identifying key pathways of epithelial dysfunction and immune activation in response to bioaerosol exposure [35–37]. These findings support targeted interventions such as advanced air filtration, optimized ventilation, antimicrobial surface coatings, and context-specific public health policies. Early detection systems also offer promise; for instance, Qiu et al. emphasize the importance of real-time airborne pathogen monitoring to guide infection control in high-risk areas like hospitals and public transport [38].

Mitigation strategies now incorporate technologies for efficient bioaerosol sampling and detection, with the goal of reducing disease spread and enabling rapid protective action [38, 39]. As we continue to understand the diverse impacts of bioaerosols on respiratory health, integrating these strategies into environmental and occupational health frameworks becomes increasingly vital.

The following sections of this article will critically discuss the underlying mechanisms by which bioaerosols influence airway epithelial integrity, activate immune responses, and contribute to disease progression. We will also highlight gaps in current knowledge and propose evidence-based strategies for exposure mitigation.

## II. Methods

A comprehensive literature review was conducted to evaluate the impact of bioaerosol exposure on airway epithelial function, mucociliary clearance, innate immune activation, and the development of chronic airway remodeling. Emphasis was placed on the interaction between microbial components (e.g., endotoxins, fungal spores, mycotoxins) and host defense mechanisms, including pattern recognition receptors, surfactant proteins, and genetic susceptibility loci. The databases PubMed and Google Scholar were searched for relevant studies published primarily between 2000 and 2025, with earlier foundational studies included where mechanistically important.

The following search terms were used alone and in combination: “bioaerosols and airway disease,” “mucociliary clearance impairment,” “aflatoxins and ciliary beat frequency,” “PRRs and TLRs in airway epithelium,” “innate immunity and fungal exposure,” “surfactant protein A and D immunity,” “asthma airway remodeling,” “cytokines and airway fibrosis,” “genetic polymorphisms TLR2 TLR4 CD14,” “NOD1 polymorphisms asthma,” “chronic inflammation and EMT,” “SP-A SP-D allergen binding,” “occupational bioaerosol exposure,” and “air pollution and innate immunity.”

Studies were filtered based on their relevance to airway pathophysiology in the context of bioaerosol exposure. Exclusion criteria included non-peer-reviewed articles, commentaries, and studies without primary data or mechanistic insight into respiratory outcomes. Priority was given to high-quality evidence including in vitro epithelial cell models, animal studies, randomized controlled trials, meta-analyses, and systematic reviews that addressed either mechanistic pathways or clinical correlations.

## III. Types of Bioaerosols

### IIIa. Fungi and Bacteria

Fungal and bacterial bioaerosols are widespread in various environments, growing on organic materials such as paper, textiles, wood, and damp surfaces. These microorganisms release allergens, enzymatic proteins, toxins, and volatile organic compounds (VOCs), which can cause toxic effects, irritations, infections, and allergic reactions [40]. Their growth is influenced by meteorological factors, particularly relative humidity, and their airborne concentrations tend to increase during monsoon seasons [41]. Common indoor reservoirs for fungal and bacterial bioaerosols include humidifiers, air conditioning systems, water-damaged carpets, showerheads, and damp ceiling panels [42].

### IIIb. Endotoxins

Endotoxins are lipopolysaccharide (LPS) from Gram-negative bacteria with high pro-inflammatory activity [43]. They consist of a core polysaccharide chain, O-specific polysaccharide side chains (O-antigen), and a lipid component (Lipid A), which is responsible for their toxic effects [44]. Due to their ability to bind easily to dust particles, endotoxins pose a constant inhalation risk [45].

Human responses to endotoxins vary based on dose, exposure route, and individual susceptibility. Symptoms include fever, shivering, increased white blood cell count (leukocytosis), neutrophilic airway inflammation, dyspnea, bronchial obstruction, and chest tightness [46]. Endotoxins have been identified as major contributors to occupational lung diseases such as organic dust toxic syndrome [47]. Occupational exposure studies report significant endotoxin levels among textile workers (2160 EU/m^3^), dairy workers (329 EU/m^3^), animal feed and grain workers (662 EU/m^3^), and sewage treatment plant workers (214 EU/m^3^) [48–50].

### IIIc. β-Glucans

β-Glucans are glucose polymers found in the cell walls of bacteria, fungi, yeasts, algae, lichens, and plants (e.g., oats and barley) [51]. Their airborne concentrations vary across different environments. In Ohio, USA, indoor β-glucan levels were 1.0 ng/m^3^ (range: 0.81–1.2 ng/m^3^), while outdoor levels were significantly higher at 7.34 ng/m^3^ (range: 6.1–8.9 ng/m^3^) [52]. In Beijing, China, concentrations ranged from 0.02 to 1.2 ng/m^3^ across multiple urban locations, including offices, hospitals, dormitories, subway stations, and commercial streets [53]. In contrast, β-glucan concentrations in concentrated animal feeding operations in Illinois, USA, were much higher, ranging from 2.4 to 538 ng/m^3^ [54].

β-Glucans have been studied for their immune-modulating properties, often being used in dietary supplements for immune enhancement and treatment of high cholesterol, diabetes, and cancer [55]. However, airborne exposure to β-glucans has been associated with inflammatory responses and respiratory symptoms, with effects varying based on glucan type and co-exposure to other bioaerosols [56].

### IIId. Mycotoxins

Mycotoxins are toxic secondary metabolites produced by fungi and molds. A single mold species can produce multiple mycotoxins, and the same mycotoxin can be produced by multiple fungal species [57]. These compounds are classified based on their chemical structures and reactive functional groups, including amines, carboxylic acids, hydroxyl groups, lactams, and amides [58].

Health effects associated with mycotoxin exposure range from immune suppression, allergic reactions, and irritation to severe diseases and even death. The severity of mycotoxicosis depends on exposure duration, concentration, and host factors such as age, health status, and genetics [59]. Additional risk factors, including alcohol consumption, vitamin deficiencies, caloric deprivation, and existing infections, can exacerbate the toxic effects of mycotoxins [59]. Mycotoxin exposure primarily occurs via ingestion, inhalation of airborne spores, and dermal contact with contaminated surfaces.

### IIIe. Allergens

Allergens are antigens that trigger abnormal immune responses, leading to allergic reactions. Symptoms of allergen exposure include runny nose, nasal congestion, sore throat, itchy eyes, and sneezing, with prolonged exposure increasing the risk of asthma and other allergic diseases. Common bioaerosol allergens include fungal spores and hyphae, arthropods (dust mites, cockroaches), vascular plants (fern spores, pollen, soy dust), pet dander, and royal jelly [60,61]. Environmental factors such as mechanical disturbances, wind, rain, and active spore discharge influence allergen release into the air. Indoor humidity and water-damaged surfaces (e.g., carpets, ceilings, walls) serve as major reservoirs for dust mite and mold allergens [62]. Improper ventilation and high indoor moisture levels further increase bioaerosol allergen concentration, exacerbating allergic diseases and asthma. Table 1 summarizes key bioaerosol types, their sources, innate immune receptors involved in host recognition, mechanisms of airway injury, and the resulting clinical manifestations such as asthma and chronic inflammation.

## IV. Acute Immune Responses to Bioaerosols

### IVa. Mucociliary Clearance

Mucociliary clearance is the primary innate defense mechanism of the lungs, responsible for removing inhaled pathogens and particles. The functional components are the protective mucous layer, the airway surface liquid layer, and the cilia on the surface of ciliated cells. It relies on ciliated epithelial cells, which move in coordinated waves to propel mucus, along with trapped foreign materials, toward the throat for elimination [63–65]. This system is significantly impaired by bioaerosols. Lee et al. demonstrate that aflatoxins, mycotoxins secreted by Aspergillus species, reduce ciliary beat frequency (CBF) in airway epithelial cells. This reduction in CBF impairs mucociliary clearance, a critical defense mechanism of the respiratory tract, thereby contributing to the pathogenesis of fungal airway diseases [66]. Thus, exposure to microbial toxins and allergens reduces ciliary beat frequency and disrupts epithelial junction integrity, impairing the migration and repair of airway epithelial cells. This dysfunction results in increased mucus retention and impaired pathogen clearance, predisposing the airways to recurrent infections and chronic inflammation. The destruction of epithelial tight junctions by proteolytic allergens further facilitates deeper bioaerosol penetration into the airway tissue, triggering sustained immune activation and local tissue damage [67].

### IVb. Innate Immune Receptors

Bioaerosols, including allergens, bacterial endotoxins, and fungal spores, interact with the airway epithelium through innate immune receptors, including Toll-like receptors (TLRs), pattern recognition receptors (PRRs), protease-activated receptors (PARs), and calcium-dependent collectins such as pulmonary surfactant proteins A and D (SP-A and SP-D), as well as mannose binding protein. These interactions activate immune pathways, leading to epithelial cell signaling, cytokine production, and recruitment of inflammatory cells like macrophages, dendritic cells, and eosinophils.

#### IVb.1 Pattern Recognition Receptors (PRRs) and Toll-Like Receptors (TLRs)

Epithelial cells serve as PRR-bearing cells, playing a role in innate immune system defense by recognizing pathogen-associated molecular patterns (PAMPs)—structural motifs unique to microbes. Among PRRs, TLRs are key receptors in microbial recognition and immune activation. Originally discovered in Drosophila, their human counterparts were identified shortly after as components of host defense against bacteria, viruses and fungi [68–70].

PRRs are classified based on their ability to detect microbial components. TLR-1, TLR-2, TLR-4, TLR-5, and TLR-6 recognize bacterial surface markers, such as lipopolysaccharides (LPS) from Gram-negative bacteria and peptidoglycans from Gram-positive bacteria. TLR-3, TLR-7, TLR-8, and TLR-9 detect viral RNA and unmethylated CpG DNA, allowing identification of viral infections [71–74]. Other PRRs include nucleotide-binding oligomerization domain (NOD1, NOD2) receptors, scavenger receptors (SR-A and SR-B), C-type lectin receptors (CLRs) (e.g., mannose and dectin-type receptors), and macrophage galactose-type lectin (MGL) receptors, all of which contribute to microbial clearance, facilitating immune modulation [75–79].

Upon activation, PRRs trigger intracellular signaling cascades that lead to three primary immune responses. First, they induce immediate antimicrobial defenses through reactive oxygen species (ROS) production, antimicrobial peptide release, and enzymatic degradation of pathogens. Second, they stimulate inflammatory cytokine production (IL-6, IL-8, TNF-α, and interferons), which recruit neutrophils, macrophages and dendritic cells to the infection site, amplifying the immune response. Finally, PRRs contribute to the initiation of adaptive immunity by enhancing antigen presentation to T-lymphocytes, which strengthens long-term immune memory and allows for a faster response to future pathogen exposure [70].

#### IVb.2 Role of SPA-A and SPA-D in Airway Immunity

SP-A and SP-D, members of the pulmonary surfactant protein family, are essential for immune defense in the airways. These proteins bind to various carbohydrates on bacterial, fungal, and viral surfaces, facilitating pathogen clearance and immune regulation [80,81]. SP-A-deficient mice exhibit delayed microbial clearance and heightened inflammation, while SP-D deficiency results in impaired viral clearance and increased airway inflammation [80,82]. In addition to their role in pathogen clearance, SP-A and SP-D modulate allergic airway inflammation. They bind to environmental allergens, including house dust mites and Aspergillus fumigatus, blocking IgE binding and thus reducing allergic inflammation [81,83,84]. These surfactant proteins also suppress lymphocyte proliferation, monocyte maturation, and IL-8 production by eosinophils, further downregulating inflammatory responses [85–87]. By directly binding to glycosylated allergen regions, SP-A and SP-D not only contribute to pathogen elimination but also help regulate immune tolerance, minimizing unnecessary airway inflammation and hyperreactivity.

## V. Chronic Inflammation and Airway Remodeling: Cytokine-Mediated Fibrosis and Airway Thickening

Prolonged exposure to bioaerosols leads to a cascade of inflammatory and structural changes within the airways, culminating in chronic inflammation, airway fibrosis, and smooth muscle hypertrophy, all of which contribute to irreversible airway remodeling and progressive lung dysfunction [88]. This process is driven by bioaerosol-induced cytokine production, particularly IL-6 and TNF-α, which promote fibroblast activation and extracellular matrix deposition, exacerbating airway fibrosis and asthma pathogenesis [89]. In chronic exposure settings, persistent inflammation induces epithelial-to-mesenchymal transition (EMT), further amplifying tissue remodeling and airway wall thickening [90–93]. Airway remodeling is marked by epithelial barrier dysfunction, goblet cell metaplasia, airway smooth muscle thickening, and increased angiogenesis, which drive steroid-resistant asthma and acute exacerbations. These structural changes increase airway stiffness and reduce bronchodilator responsiveness, hallmarks of severe asthma and COPD [94–96].

## VI. Genetic Polymorphisms and Susceptibility to Asthma

Asthma has an estimated heritability of 60%-80%, with genome-wide association studies (GWASs) identifying 128 asthma-associated single-nucleotide polymorphisms (SNPs), primarily in European populations [97]. However, populations with a high disease burden, such as those of African ancestry, have been underrepresented, prompting efforts to increase genetic research in diverse populations [98–100]. Chang et al. conducted the largest meta-analysis of genetic variation in asthma among African American individuals, starting with GWASs in 6975 asthma cases and 4429 controls at CHOP [101]. While individual cohort analyses showed no significant results, combining the three datasets identified a novel locus on chromosome 6 of unknown functional significance [101]. A subsequent meta-analysis of 19,628 subjects across 13 datasets revealed 12 loci meeting genome-wide significance [98,99,101].

Additionally, genetic polymorphisms in TLR2, TLR4, and CD14 influence susceptibility to asthma. Variations in these genes modulate immune responses to bioaerosols, shaping disease severity [88]. Polymorphism for CD14 was shown to be associated with sCD14, total IgE and skin tests . Similarly, a polymorphism in TLR gene was a major susceptibility gene for children living on farms. Eder et al. identified TLR2 as a major gene for asthma in children of European farmers, with the TLR2/-16934 T allele being associated with a lower risk of asthma and atopic sensitization among farm children [102]. Lau et al. found significant interactions between TLR6 SNPs and childhood farm exposure, suggesting that genetic variations in TLR genes modulate the protective effects of farm environments on asthma risk [103]. Polymorphisms of intracellular NOD1 protein, which binds cell wall peptidoglycans of Gram-negative bacteria, were shown to be especially associated with atopic eczema and asthma

## VII. Challenges and Considerations in Bioaerosol Monitoring and Exposure Assessment

### VIIa. Bioaerosol Sampling and Exposure Assessment

Bioaerosol sampling has gained significant attention as a tool for assessing occupational exposures, identifying potential health hazards, and monitoring the transmission of infectious diseases in healthcare and industrial settings [5,104,105]. However, bioaerosol monitoring presents several challenges, particularly due to the lack of standardized exposure limits for airborne bioaerosols such as fungi, bacteria, pollen, and viruses [23,104,106–108]. While exposure limits exist for certain organic materials like cotton and grain dust, no definitive concentration threshold has been established for bioaerosols due to their complex nature, diverse health effects, and inter-individual variability in immune response [109]. Moreover, bioaerosols can cause respiratory illnesses through multiple pathways, including allergic reactions, infections, and toxicity, further complicating exposure assessments [104,110].

### VIIb. Bioaerosols in Disease Transmission and Environmental Influences

Bioaerosols play a critical role in disease transmission, particularly in healthcare settings, where infectious microorganisms such as measles and tuberculosis can be aerosolized and spread via respiratory droplets [111,112]. Occupational exposure to soil saprophytic fungi, such as Coccidioides immitis, can also pose a risk, especially in settings where soil disturbances lead to aerosolization and subsequent inhalation, potentially resulting in acute pulmonary infections [113,114]. Beyond healthcare, bioaerosol concentrations fluctuate significantly based on environmental conditions, with studies showing that fungal and bacterial bioaerosol levels vary seasonally. For example, bacterial bioaerosol concentrations in Tehran, Iran, were significantly higher in summer (1973 CFU/m^3^) compared to winter (1016 CFU/m^3^) [115], while in the USA, winter fungal concentrations ranged from 3 to 59 CFU/m^3^ and bacterial levels varied from 19 to 607 CFU/m^3^ [42]. In South Korea, fungal bioaerosol concentrations increased from 24–654 CFU/m^3^ in winter to 60–930 CFU/m^3^ in summer, indicating the influence of temperature and humidity on bioaerosol proliferation [66,116].

### VIIc. Indoor Bioaerosol Concentrations and Influencing Factors

Indoor bioaerosol concentrations are highly dependent on occupancy and ventilation. Xin et al., 2021 conducted a study at Universiti Sains Malaysia, assessing the density and diversity of airborne fungi. They found that indoor fungal concentrations ranged from 81 to 1743 CFU/m^3^ in Trial 1 and from 229 to 699 CFU/m^3^ in Trial 2, with predominant genera being Aspergillus, Penicillium, and Cladosporium [117]. Similarly, bioaerosol concentrations in laboratories (320 and 460 CFU/m^3^ for bacteria and fungi, respectively) were higher than those in office rooms (61 and 140 CFU/m^3^), demonstrating how occupant density influences microbial loads [118]. Poor ventilation has also been shown to increase indoor bacterial bioaerosol levels, particularly in enclosed spaces [13]. In healthcare settings, infectious bioaerosol concentrations decline with increasing distance from an infected patient, as larger droplets settle out of the air [112]. These spatial variations in bioaerosol distribution emphasize the need for careful sampling strategies to accurately assess exposure risks [115,119].

### VIId. Temporal Variability and the Need for Comprehensive Monitoring

Because bioaerosol concentrations fluctuate with time and environmental factors, their monitoring should be part of a comprehensive exposure assessment strategy, integrating environmental inspections, HVAC assessments, and health surveys [120–122]. Temporal variations have been observed in multiple studies, such as indoor airborne mold levels being 26 times higher in summer than in winter [123] and fungal bioaerosols exhibiting daily fluctuations, with higher concentrations in the morning than in the afternoon [119]. Similarly, in a study on airborne influenza virus in a healthcare clinic, bioaerosol concentrations varied daily depending on patient presence and viral shedding [124]. These findings highlight the limitations of single-timepoint bioaerosol sampling and the importance of longitudinal monitoring across multiple locations and time periods [108].

Despite these challenges, bioaerosol sampling remains an essential tool for research and exposure assessment when used appropriately. It is particularly useful for comparing indoor and outdoor bioaerosol levels to identify problem sources or for targeting specific microbial contaminants in occupational settings such as composting operations or sewage treatment facilities [125,126]. However, routine bioaerosol sampling without a clear interpretation strategy can be misleading, given the lack of established dose-response data and inherent variability in bioaerosol exposure. As a result, agencies such as NIOSH recommend environmental inspections and remediation efforts over routine air sampling for assessing respiratory illness risks in damp buildings [108].

Overall, while bioaerosol sampling remains a crucial component of exposure assessment, its effectiveness relies on thoughtful application and interpretation within a broader environmental and occupational health framework. Future research and standardized guidelines are needed to enhance its utility in mitigating health risks associated with airborne microbial contaminants.

## VIII. Strategies to Control and Mitigate Bioaerosol-Related Health Effects

### VIIIa. Source Control

Source control is a primary strategy for mitigating bioaerosol-related health effects. This includes reducing resuspension of particles, implementing integrated pest management, and controlling moisture levels to prevent mold growth. Matsui et al. highlighted the effectiveness of IPM practices, such as sealing entry points, reducing food and water sources, and using non-chemical control methods, in reducing indoor allergens and bioaerosols associated with asthma exacerbations [127]. Wu and Wong emphasized the importance of controlling indoor relative humidity levels to prevent mold growth, recommending maintaining RH levels below 75% or at 30–60% to prevent mold contamination [128]. Regular cleaning and maintenance of indoor environments are essential. Periodic cleaning operations, maintenance activities, and proper waste management can reduce the accumulation of bioaerosols [129]. Additionally, controlling sources of bioaerosols in specific environments, such as wastewater treatment plants, involves using aeration-based strategies, improving ventilation, and implementing protective measures such as periodic monitoring of disinfection efficiency and pathogenic load. [130–132].

### VIIIb. Ventilation Improvements

Enhancing ventilation is another critical strategy. Ensuring sufficient ventilation in buildings can significantly reduce bioaerosol concentrations. This can be achieved by increasing the rate of air exchange and using high-efficiency particulate air (HEPA) filters in HVAC systems. HEPA filters are particularly effective in capturing airborne particles, including bioaerosols, thereby improving indoor air quality. The American Society of Heating, Refrigerating and Air-Conditioning Engineers (ASHRAE) recommends maintaining proper ventilation to reduce indoor bioaerosol levels [133]. Improved ventilation strategies, such as effective ventilation with adequate supply of clean air and minimizing air recirculation, are crucial in specific settings like wastewater treatment plants [129,134].

### VIIIc. Air Filtration and Disinfection

Air disinfection methods, such as germicidal ultraviolet (UV) light, can further reduce the presence of bioaerosols. UV light has been shown to inactivate various microorganisms, including bacteria and viruses, making it a valuable tool in mitigating bioaerosol-related health risks. Lu et al. evaluated the performance of UV disinfection across the 222–365 nm spectrum against aerosolized bacteria and viruses, including Escherichia coli, Staphylococcus epidermidis, Salmonella enterica, MS2, P22, and Phi6. They found that the krypton chloride excilamp emitting at 222 nm was the most efficient in inactivating viral bioaerosols, while a low-pressure mercury lamp emitting at 254 nm performed well in both inactivation efficacy and energy efficiency [135]. Additionally, air filtration systems equipped with HEPA filters can effectively remove bioaerosols from indoor air [136]. The use of tight-fitting face masks to trap infectious aerosols and reduce inhalation exposure to contaminated air is also critical for disease control [39,134,137].

### VIIId. Layered Approach

Implementing these strategies in a layered approach maximizes their effectiveness. Combining source control, ventilation improvements, air filtration, and disinfection methods can significantly reduce bioaerosol concentrations and mitigate their health effects. This approach ensures a comprehensive management of bioaerosol exposure, leading to safer indoor environments and improved public health outcomes [39,129,130]. Layered intervention strategies are needed to maximize risk reduction, as demonstrated in case studies.

#### Monitoring and Maintenance

Continuous monitoring and maintenance of air quality systems are essential to ensure their effectiveness. Regular inspection and maintenance of HVAC systems, air filters, and disinfection devices help maintain optimal performance and prevent the buildup of bioaerosols. A systematic review and meta-analysis by Dai et al. demonstrated that HVAC systems, particularly those equipped with HEPA filters, effectively reduce bioaerosol concentrations in hospital environments. The study found that regular maintenance of these systems is crucial for their optimal performance and effectiveness in removing airborne bacteria and fungi [136]. Recent progress in sensor-based data collection, analysis, cloud-based storage, and early warning techniques in wastewater treatment plants may help reduce the risk of infectious transmission, especially during a pandemic situation. Zhao et al. developed and evaluated early-warning methods based on wastewater surveillance data in Detroit, Michigan. They designed eight early-warning methods and demonstrated their utility in providing early warnings for COVID-19 incidences, with hit rates reaching up to 100%. These methods assist health departments in assessing trends and implementing quick public health responses [138]. Additionally, Parkins et al. discuss the transformative potential of wastewater-based surveillance (WBS) for public health action. They highlight how novel data-sharing tools have enabled real-time, cost-effective, and comprehensive monitoring of SARS-CoV-2 RNA in wastewater, providing early warnings of COVID-19 surges and facilitating evidence-informed decision-making [139]. Table 2 outlines common occupational and environmental sources of bioaerosol exposure, the associated microbial components, their relative risk levels, health consequences, and recommended mitigation strategies.

By addressing these key areas, effective strategies can be developed to control and mitigate the health effects of bioaerosols, ensuring safer indoor environments and improved public health outcomes.

## IX. Conclusion

Bioaerosols play a multifaceted role in the development and progression of airway diseases through mechanisms involving epithelial dysfunction, innate and adaptive immune activation, and chronic inflammation. Genetic susceptibility and environmental variability further modulate individual responses to exposure. Despite ongoing challenges in exposure assessment and standardization, integrated strategies including source control, ventilation, filtration, and real-time monitoring offer promising ways to mitigate health risks. Continued interdisciplinary research is essential to refine our understanding and guide targeted interventions that protect respiratory health in both clinical and public health settings.

## Figures and Tables

**Figure 1:**
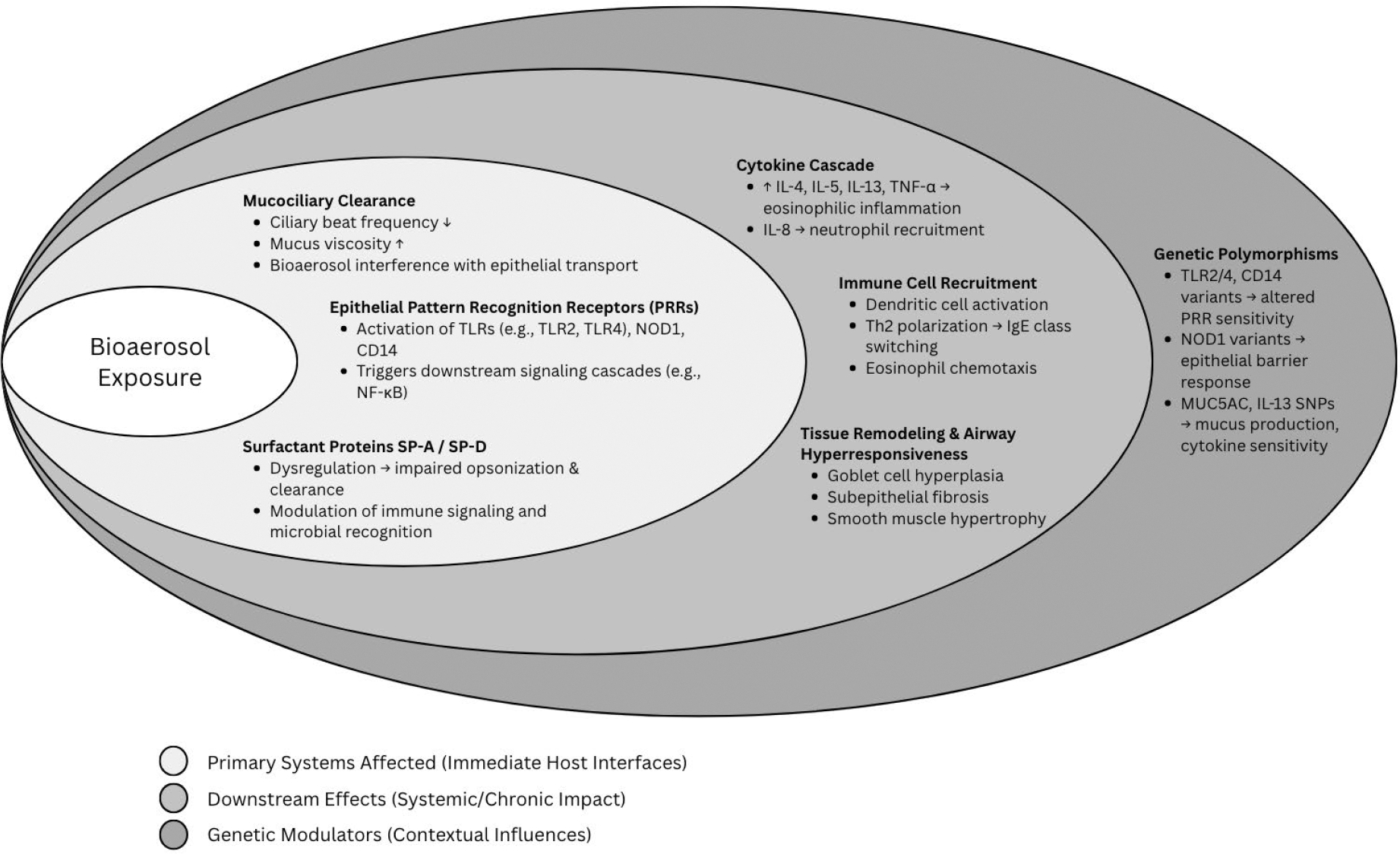
Circular Systems Map Illustrating Epithelial, Immune, and Genetic Interactions in Bioaerosol-Induced Asthma.

**Table 1:** Bioaerosol Components and Their Pathophysiologic Mechanisms.

Bioaerosol Component	Source	Innate Immune Receptors	Mechanism of Injury	Clinical Outcomes
Endotoxins (LPS)	Gram-negative bacteria	TLR4, CD14	Cytokine storm, barrier dysfunction	Asthma, COPD exacerbation
β-Glucans	Fungal cell walls	Dectin-1, TLR2	Th2/Th17 skewing, immune activation	Allergic asthma, hypersensitivity
Mycotoxins	Fungal spores	N/A (toxic effect)	Epithelial injury, oxidative stress	Chronic inflammation, carcinogenesis
Allergens (e.g., pollen)	Plants, fungi, insects	TLR4, TLR9, SP-A/SP-D (modulate)	IgE-mediated hypersensitivity	Allergic rhinitis, asthma
Bacteria/Fungi	Soil, compost, water, etc.	TLR2, TLR4, TLR5, NOD1	Pattern recognition, cytokine release	Non-atopic asthma, hypersensitivity pneumonitis

**Table 2:** Environmental Exposure Sources and Associated Bioaerosol Risks.

Exposure Source	Common Bioaerosols	Risk Level	Associated Health Outcomes	Mitigation Strategies
Wastewater treatment plants	Bacteria, endotoxins, fungi	High	Asthma, chronic bronchitis	PPE, closed systems, ventilation
Agricultural composting	Fungi, β-glucans, spores	High	Hypersensitivity pneumonitis, asthma	Mask use, HEPA filtration, limited exposure time
Indoor damp environments	Mold, mycotoxins	Moderate - High	Allergic asthma, cough, wheezing	Dehumidification, mold remediation, air filters
Urban pollution	Mixed allergens, particulate - bound	Moderate	Atopic disease, asthma	Air quality monitoring, indoor air purifiers
Food processing facilities	Organic dust, bacteria	Moderate	Rhinitis, asthma	PPE, hygiene practices, improved airflow
